# Arthroscopic Treatment of Chronic Acromioclavicular Dislocation With Semitendinosus Autograft and Coracoclavicular Suspension Fixation

**DOI:** 10.1016/j.eats.2022.06.014

**Published:** 2022-09-17

**Authors:** Pablo Cañete San Pastor, Inmaculada Prosper Ramos, Javier Lopez Valenciano, Ivan Copete

## Abstract

The management of acromioclavicular dislocations remains controversial. On many occasions, these chronic dislocations are asymptomatic. However, there are patients who, despite good rehabilitation treatment, do present with pain, periscapular muscle fatigue, weakness, paresthesia or scapular dyskinesia. In these patients, surgical treatment is indicated.

Despite being a frequent injury (12% of shoulder injuries and up to 40% in contact athletes), the management of acromioclavicular (ACC) dislocations remains a controversial issue.^1^ This variability in the management of ACC dislocations means that we find chronic type III, IV, or V ACC dislocations relatively frequently (>3 or 6 weeks of evolution, according to different authors).^2^, ^3^, ^4^, ^5^ On many occasions, these chronic dislocations will not be symptomatic or will improve with adequate rehabilitation treatment and will not require surgical treatment. However, there are patients who, despite good rehabilitation treatment, present with pain, periscapular muscle fatigue, weakness in the affected arm, paresthesia, or scapular dyskinesia. It is in these patients in whom surgical treatment of chronic ACC dislocation is indicated.^6^^,^^7^

There are studies that show better results if vertical and horizontal anatomical restoration of the ACC joint is achieved after a dislocation. This principle is valid for both acute and chronic dislocations.^8^

Surgical treatment in the chronic phase is different from that in the acute phase. Before 3 weeks, we consider that the coracoclavicular (CC) and ACC ligaments have healing capacity, so our treatment will basically be reduction and mechanical stabilization that allows the ligaments to heal as anatomically as possible. However, in chronic cases, degeneration and atrophy of the CC and ACC ligaments mean that we must add biological augmentation in addition to mechanical fixation. For this biological augmentation, the most used options are coracoacromial ligament transfer or a tendon graft (allograft or autograft).^9^, ^10^, ^11^, ^12^, ^13^, ^14^, ^15^

Our treatment of choice in cases of chronic (>3 weeks) symptomatic ACC dislocation and after failure of orthopaedic treatment is arthroscopy-assisted ACC and CC ligamentous reconstruction with semitendinosus autograft and mechanical fixation with a CC suspension device using the ZipTight fixation system (Zimmer Biomet, Warsaw, IN) to restore vertical and horizontal anatomical stability. In Table 1, we list the advantages and disadvantages of this technique.

**Table 1:** Advantages and Disadvantages of the Technique

Advantages	Disadvantages
Biological reconstruction of the coracoclavicular and acromioclavicular ligaments	Demanding technique
Horizontal and vertical stability	Comorbidity of the semitendinosus autograft
Mechanical stabilization during biological healing process	
Small bone holes	Open access to the acromioclavicular joint
Just one hole in coracoid	
No screws	
Advantages of arthroscopic technique	

## Surgical Technique (With Video Illustration)

**Fig 1:**
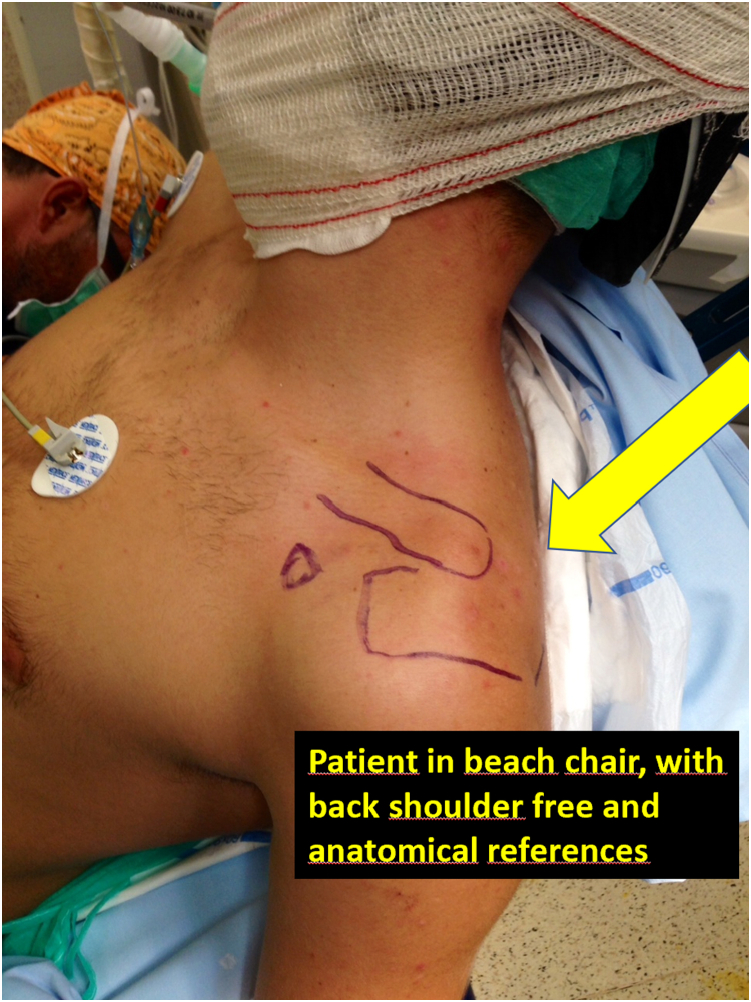
Positioning the patient semisitting and marking the anatomical references. This surgical procedure is realized with the patient in beach-chair position, with enough room on the back of the shoulder to perform an arthroscopy.

**Fig 2:**
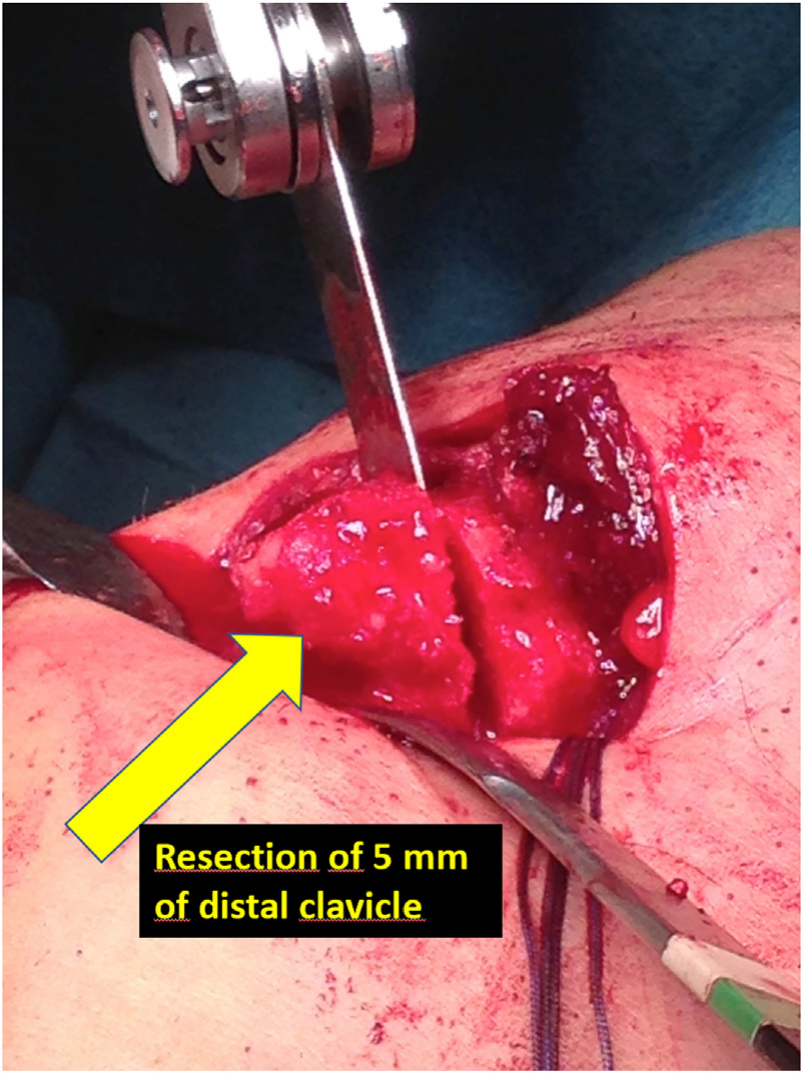
Resection of 4 to 5 mm of the distal clavicle. The patient is in the beach-chair position. After open approach to the acromioclavicular joint, a 5 mm of distal clavicle bone is resected with a saw and any meniscal tissue is removed.

**Fig 3:**
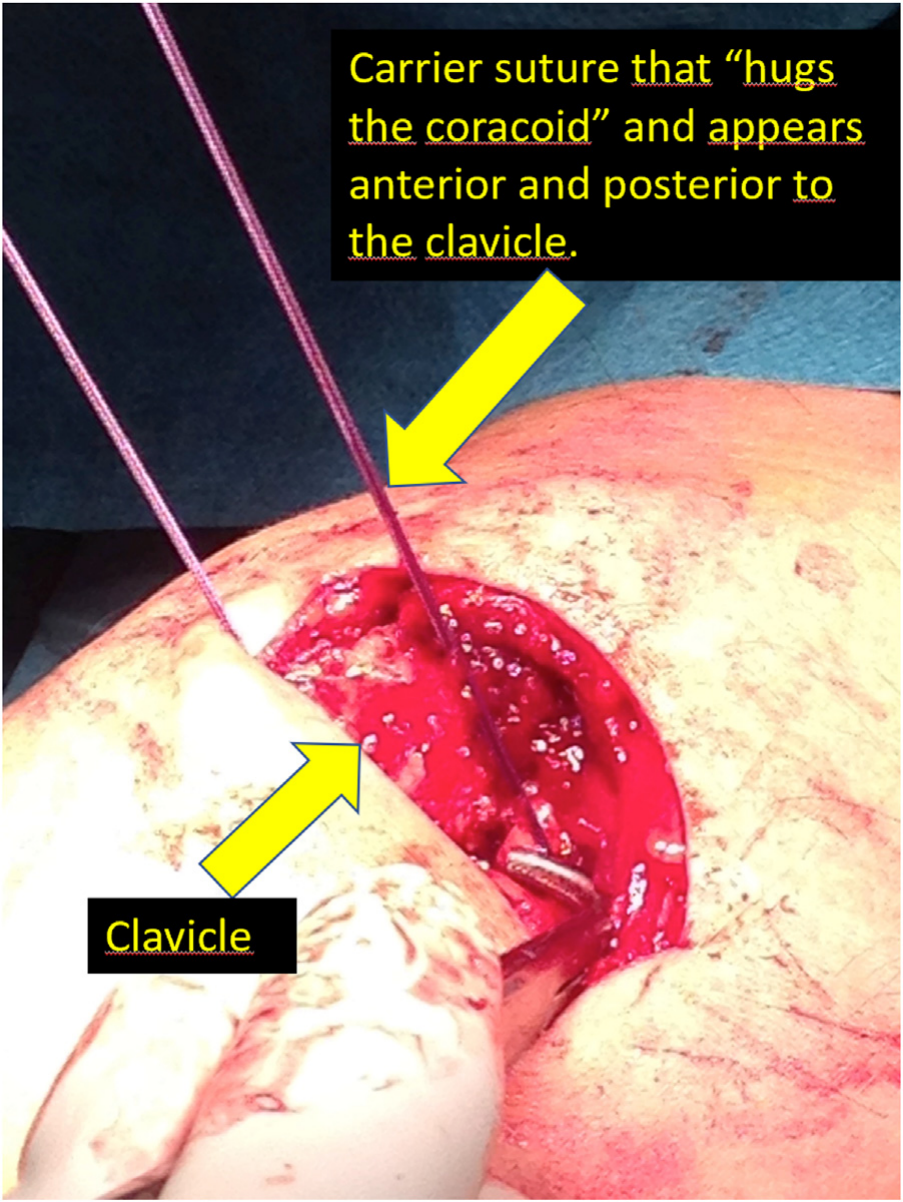
Carrier suture that “hugs the coracoid” and appears anterior and posterior to the clavicle. Using an eyelet needle and under arthroscopic control with the scope in the lateral portal, we pass a suture from the anterosuperior part of the clavicle to the inferomedial part of the coracoid. We pass another suture from the posterosuperior part of the clavicle to the inferolateral part of the coracoid. We joint both sutures and obtain a suture that goes from the posterosuperior part of the clavicle, embracing the coracoid at its lower part and again to the clavicle, at its anterosuperior surface. We use this suture as a carrier for the semitendinosus graft.

**Fig 4:**
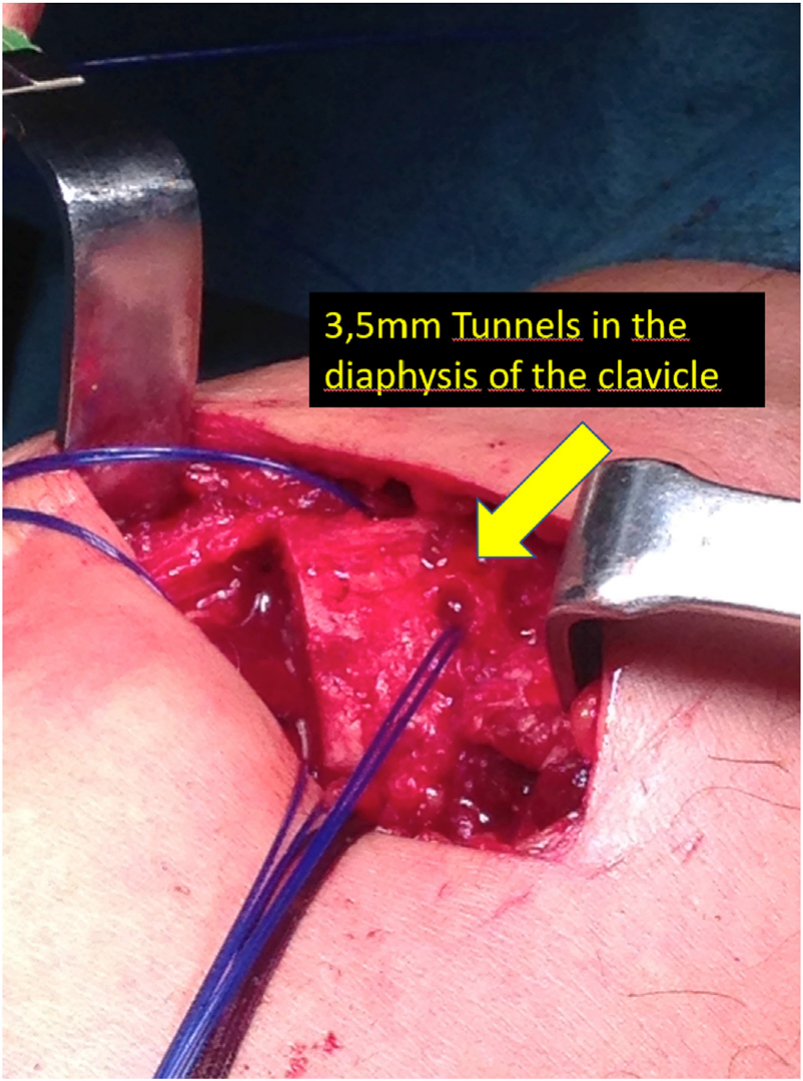
Realization of the tunnels in the diaphysis of the clavicle. We make a blind tunnel at the lateral border of the clavicle and another at the superior border of the clavicle with a 3.5-mm drill bit. We communicate both tunnels with a bone clamp, and we pass a carrier suture that we will use to pass the graft.

**Fig 5:**
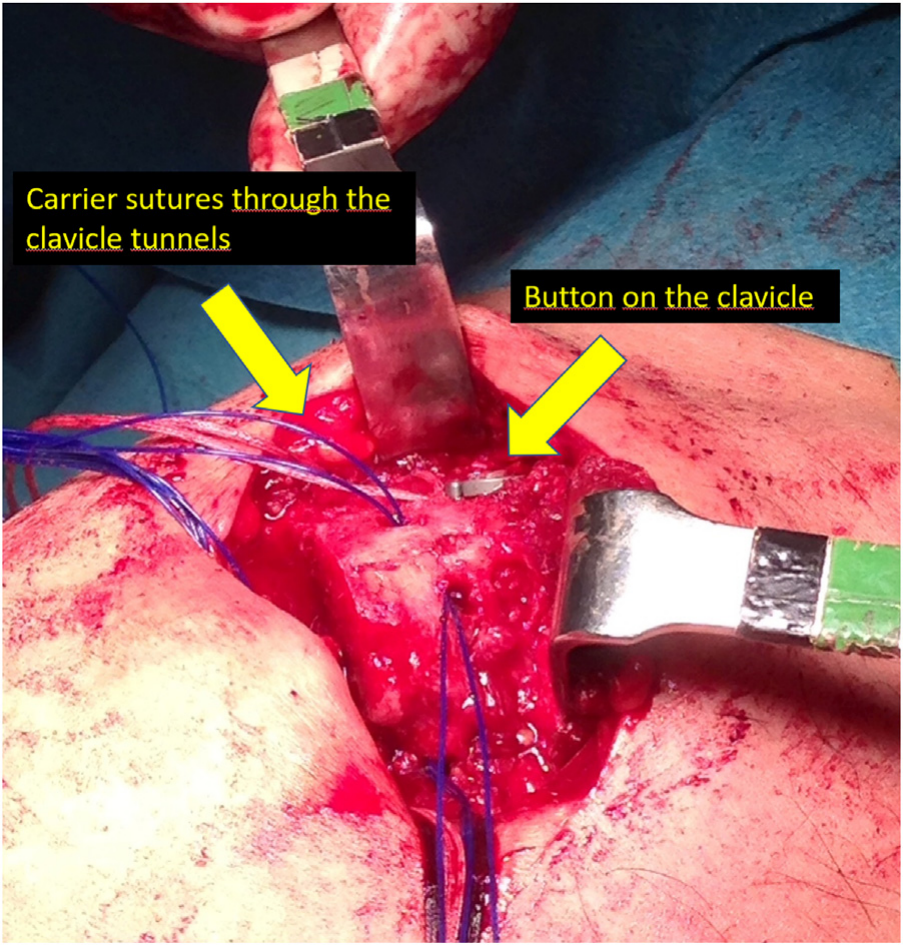
Carrier sutures through the clavicle tunnels; we will use these sutures to pass the graft across the tunnels. The clavicle button of the coracoclavicular suspension system is visible in the superior part of the diaphysis of the clavicle.

**Fig 6:**
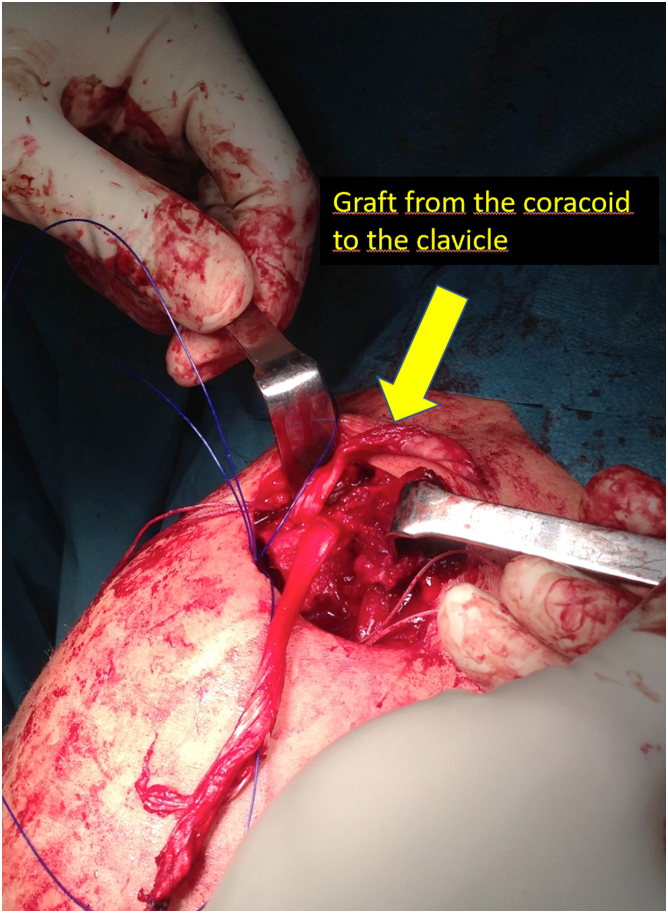
Passage of the graft from the coracoid to the clavicle. We introduce the end of the graft that comes from the lateral part of the coracoid toward the back of the clavicle through the lateral clavicle tunnel to the top of the clavicle. We then tighten the graft and suture it together. Finally, we reconstruct the coracoclavicular ligaments.

**Fig 7:**
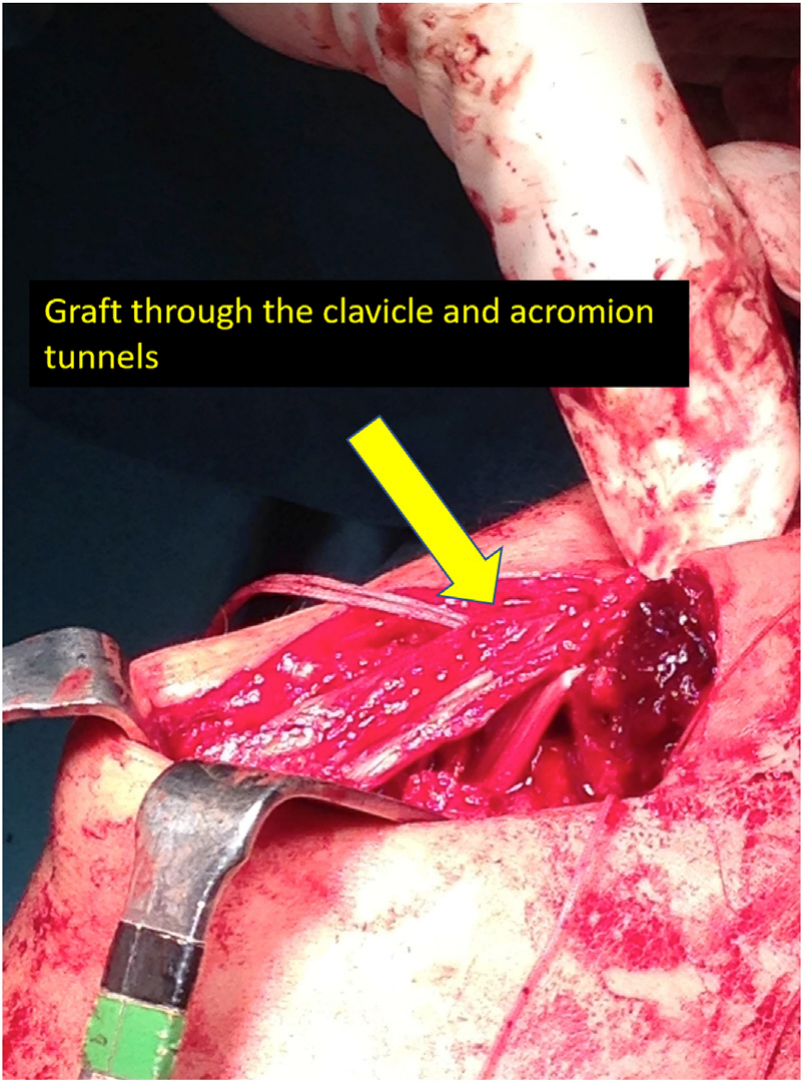
Passage of the graft through the clavicle and acromion tunnels, reconstructing the coracoclavicular and acromioclavicular ligaments. The graft is inserted and fixed in the upper part of the clavicle. Then, we pass the graft through the acromion tunnels and reconstruct the acromioclavicular ligament.

**Fig 8:**
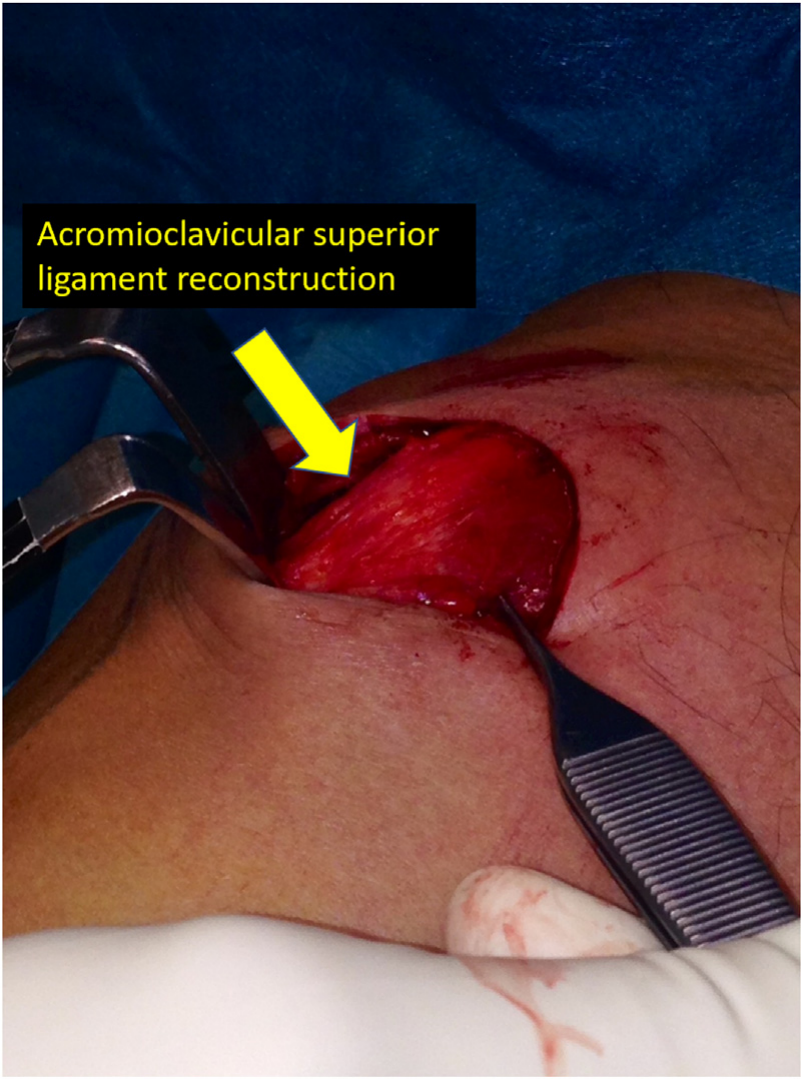
Final image of the acromioclavicular stabilization and reconstruction surgery: The coracoacromial and the superior acromioclavicular ligaments to stabilize the acromioclavicular joint in the vertical and horizontal plane.

**Fig 9:**
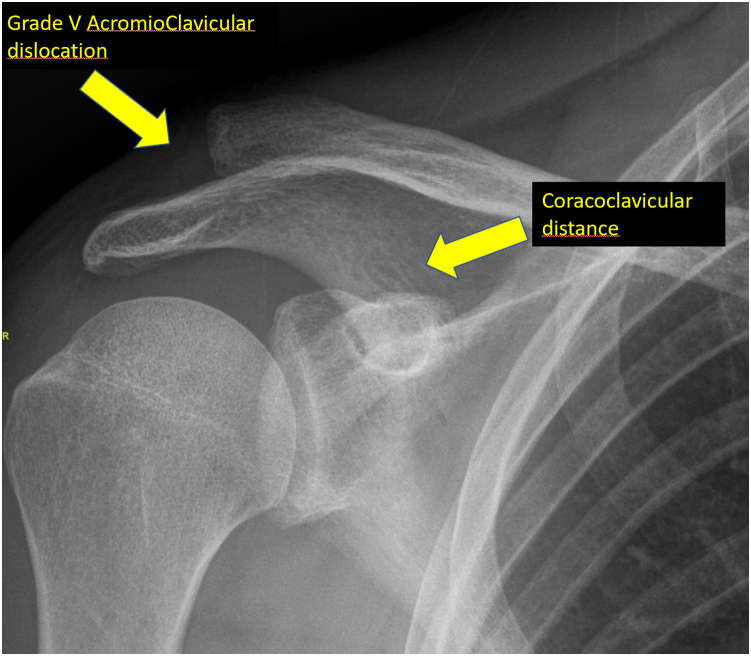
Preoperative radiograph of a patient with grade V acromioclavicular dislocation. There is no contact between the acromion and the clavicle and a large coracoclavicular distance.

**Fig 10:**
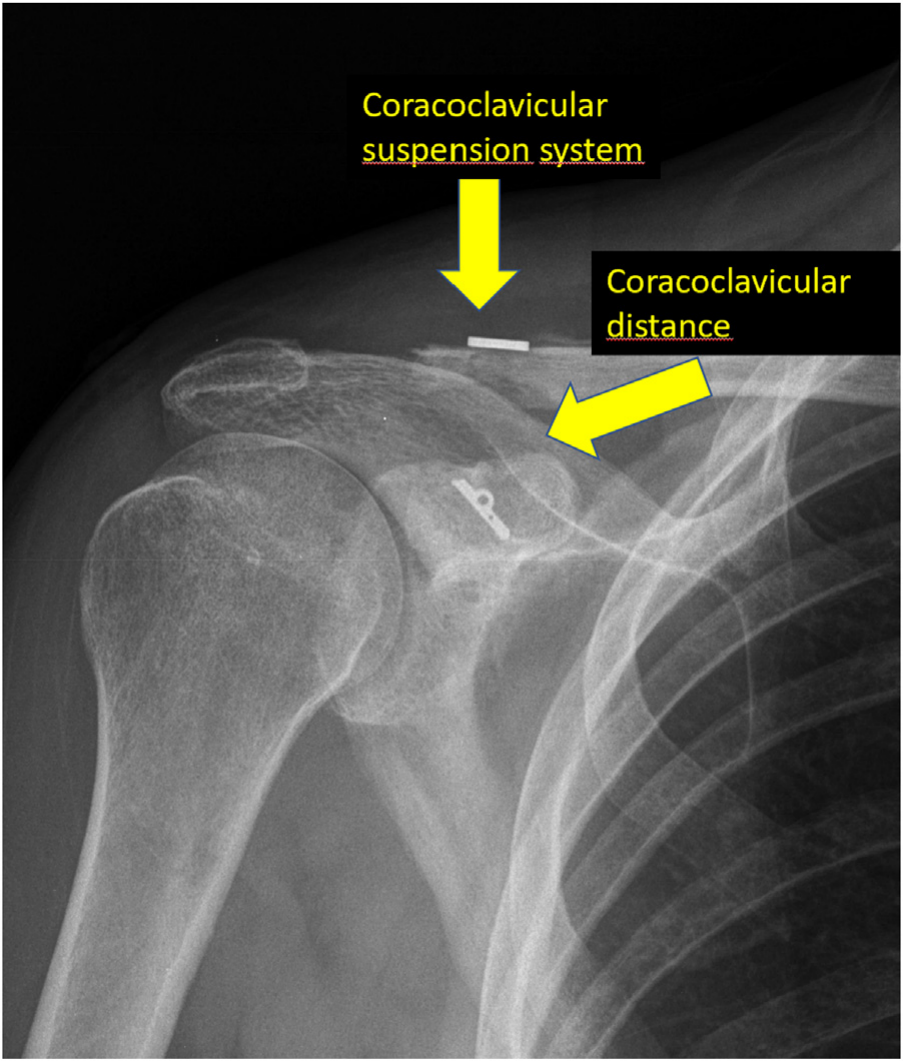
Postoperative control radiograph with decrease of coracoclavicular distance and increase of acromioclavicular distance due to the resection of 4 to 5 mm of the distal clavicle.

The surgical technique (Video 1 and Fig 1, Fig 2, Fig 3, Fig 4, Fig 5, Fig 6, Fig 7, Fig 8, Fig 9, Fig 10) is a modification of the one presented by Carofino and Mazzocca,^16^ without making tunnels in the clavicle for the passage of the graft and adding a CC fixation with a suspension system for the mechanical stabilization of the joint. The surgery is performed under plexus and general anesthesia, with the patient in a beach-chair position (Fig 1), with a 75° inclination and with axial traction during the first phase of the surgery. This traction is performed with a skin traction system with a 5-kg weight on the distal edge of the surgical table; or, in recent cases with the “trimano” positioning system. Traction is removed at the time of ACC reduction.

First, the semitendinosus autograft is taken from the ipsilateral knee with a posterior minimally invasive approach. After obtaining the graft, it is prepared on an auxiliary table, removing the muscle remains, and placing a high-strength Krakow-type suture at each end of the graft. The graft is calibrated and left wrapped in physiological serum.

Shoulder arthroscopy is performed. We begin with the standard posterior portal for evaluation of the glenohumeral joint. An anterior portal is made that is a bit lower and medial than usual to be more comfortable for the introduction of the CC guide that we will use to make the bone tunnels.

Once we have explored, reviewed, and treated the glenohumeral joint, the subacromial space is explored. After evaluation and treatment of associated pathology, if any, the coracoacromial ligament is located in the anterior part of the acromion. This ligament will be the guide that takes us to the coracoid. Once the base of the coracoid is located, we put the scope in the lateral portal and through the anterior portal we introduce the CC guide. In our case, it is an anterior cruciate ligament tibial guide, modified to have greater angulation, which allows it to be placed on the anterior face of the shoulder, from the upper surface of the clavicle to the lower part of the coracoid. We leave the guide supported on the base of the coracoid and proceed with the open surgery phase.

We make a longitudinal incision of approximately 6 to 7 cm in the upper part of the clavicle. We open the fascia and deltoid longitudinally and dissect the clavicle from about 3 cm medial to the ACC joint and about 2 cm from the acromion. We resect with an oscillating saw approximately 4 mm of the distal clavicle and remnants of the meniscus if they still exist (Fig 2).

We return to arthroscopic surgery. With the tip of the guide at the base of the coracoid, centered on the medial lateral plane, we place the other end of the guide on the upper part of the clavicle, approximately 3 cm medial to the acromial edge and centered on the anteroposterior plane. We make a hole with the guidewire and after checking the correct position in the clavicle and coracoid, we drill a tunnel through the 2 cortices of the clavicle and the 2 of the coracoid with a 4.5-cm cannulated drill. Through the cannulated drill, we pass a carrier suture that we use to pass the CC suspension implant ZipTight fixation system (Zimmer Biomet) from the top of the clavicle to the bottom of the coracoid base. We check that the metal tablet is well seated at the base of the coracoid. We place the locking button through the loop of the sutures in the upper part of the clavicle, remove the percutaneous axial traction, reduce the clavicle in both planes, with the help of a periostotome, and pull the sutures. The system locks itself, maintaining the reduction achieved.

Once we have the ACC joint reduced and mechanically fixed with the cortical suspension system, we proceed to add the biological part to the reconstruction. Using an eyelet needle and under arthroscopic control with the scope in the lateral portal, we pass a suture from the anterosuperior part of the clavicle to the inferomedial part of the coracoid. We retrieve this suture from the anterior portal. We pass another suture in the same manner from the posterosuperior part of the clavicle to the inferolateral part of the coracoid. We join both sutures and obtain a suture that goes from the posterosuperior part of the clavicle, embracing the coracoid at its lower part and again to the clavicle, at its anterosuperior surface (Fig 3). We use this suture as a carrier for the semitendinosus graft. We make a blind tunnel at the lateral border of the clavicle and another at the superior border of the clavicle with a 3.5-mm drill bit. We communicate both tunnels with a bone clamp (Figs 4 and 5). We introduce the end of the graft that comes from the lateral part of the coracoid toward the back of the clavicle through the lateral clavicle tunnel to the top of the clavicle (Fig 6). We tighten the graft well and suture it together. We have achieved the reconstruction of the CC ligaments (conoid and trapezoid) (Fig 7). With the rest of the graft that we have and that is inserted and fixed in the upper part of the clavicle, we reconstruct the ACC ligament. We made a tunnel in the acromion with the 3.5-mm drill, from the medial acromion and to its upper part, approximately 2 cm lateral to the clavicular edge of the acromion. We pass one of the ends of the graft through the acromial tunnel from medial to superior and suture it on itself, applying tension and reducing the clavicle in the horizontal plane. Thus, we achieve horizontal stability with the reconstruction of the ligaments and the superior ACC capsule (Fig 8).

The rest of the graft is sutured on itself, increasing the thickness, stability, and resistance of the ACC joint. Finally, it is important to perform a good closure of the fascia and muscle plane (Table 2).

**Table 2:** Tips and Tricks of the Technique

Create a good anterior portal thinking that we will use it to introduce the guide to the coracoid: more medial and inferior than usual
We need at least 15 mm of graft to reconstruct coracoclavicular and acromioclavicular ligaments
Do not reduce entirely the acromioclavicular joint until passing the graft; it is easier passing the graft with a dislocated acromioclavicular because one will have more space
Make a good suture of the graft together to increase the stability of the reconstruction and avoid loss of tension of the graft

### Postoperative Management

We perform a postoperative control radiograph (Figs 9 and 10). We immobilize the patient in a sling for 6 weeks so that he or she can remove it for meals, personal hygiene, and take advantage of it to perform elbow flexion and extension.

At 3 weeks, passive and self-assisted shoulder mobility begins. At 6 weeks, active mobility. At 10 weeks, strengthening of the rotators and stabilizers of the scapula begins with elastic bands. Weight-bearing takes up to 3 months to complete. Return to work is allowed after 3 months (depending on the type of work) and risky or contact sports activity not before 6 months.

## Discussion

The treatment of ACC dislocations remains a controversial issue, both acute and chronic. Although there is consensus on the orthopaedic indication of grade I and II acute dislocations, and on the surgical treatment of grade VI, controversy exists regarding the treatment of grades III, IV, and V, with authors reporting good results with conservative treatment and others who advocate surgery.^3^, ^4^, ^5^, ^6^, ^7^^,^^17^, ^18^, ^19^

We would speak of chronic ACC dislocation when faced with a dislocation that has not received surgical treatment and there is still a CC and/or ACC displacement, or in the event of a surgical treatment failure in the acute phase. On many occasions, these chronic dislocations are not going to be symptomatic. However, some patients, especially manual workers or athletes, do have pain, weakness, fatigue, or scapular dyskinesia. It is in these patients that surgical treatment of chronic ACC dislocation is indicated.^6^^,^^7^^,^^20^^,^^21^

There are multiple published surgical techniques with good results, with both open and arthroscopic surgery, anatomical or nonanatomical techniques, with autograft or allograft. What does seem clear is that better results are obtained if the vertical and horizontal anatomical restoration of the ACC joint are achieved.^8^^,^^21^, ^22^, ^23^, ^24^, ^25^

In 2021, Boileau et al.^6^ defended arthroscopic treatment for CC reconstruction in chronic dislocations since excellent results are obtained and it also allows intra-articular injuries to be treated, which they found in 48% of cases. In total, 88% of patients maintained the reduction achieved in surgery. An interesting finding of this same article is that secondary displacements occur in the first 6 months, with which it can be concluded that at 6 months the reconstruction has healed. In our series of cases, the follow-up is 1 year, and we can consider it sufficient to conclude that the radiologic results will be maintained over time.

The mechanical contribution of the button suspension system allows the clavicle to be reduced and stabilized^25^^,^^26^ to the coracoid during the autograft-healing process. In a meta-analysis in 2022, Chang et al.^26^ conclude that CC reconstruction provided more stability and resistance to deformation forces than the Weaver-Dunn procedure in the treatment of chronic ACC dislocations.^26^ We achieved an anatomical reduction. The biological contribution of the autograft allows these results to be maintained over time by healing the autograft and avoiding stress failure of the implant over time. In addition, the fact of it being an arthroscopic technique has advantages: we can diagnose and treat other injuries, we respect the soft parts and tissues more, and the size of the scar and the aesthetic result are better.

As Ruiz Ibán et al.^22^ point out, the good results obtained with the management of chronic ACC dislocations with techniques that carry out ligamentous reconstruction with graft can make us increase conservative treatment in acute dislocations, since in case of failure, we can obtain excellent results with treatment in the chronic phase. Even if there is some displacement after the surgery, we can obtain good results: Lamplot et al.,^24^ in a revision of 88 patients, concluded that radiographic loss of reduction did not correlate with return to preinjury activity level.

## Conclusions

Arthroscopic treatment for ACC and CC ligamentous reconstruction with semitendinosus autograft and mechanical fixation with a CC suspension system to restore vertical and horizontal anatomical stability can have excellent clinical and radiologic results.

## Supplementary Data

ICMJE author disclosure forms

Video 1 The surgery is performed under plexus and general anesthesia, with the patient in a beach-chair position, with a 75° inclination and with axial traction during the first phase of the surgery. Shoulder arthroscopy is performed. We begin with the standard posterior portal for evaluation of the glenohumeral joint. An anterior portal is made that is a bit lower and medial than usual to be more comfortable for the introduction of the coracoclavicular guide that we will use to make the bone tunnels. Once we have explored, reviewed, and treated the glenohumeral joint, the subacromial space is explored. After evaluation and treatment of associated pathology, if any, the coracoacromial ligament is located in the anterior part of the acromion. This ligament will be the guide that takes us to the coracoid. Once the base of the coracoid is located, we put the scope in the lateral portal and through the anterior portal we introduce the coracoclavicular guide. In our case, it is an anterior cruciate ligament tibial guide, modified to have greater angulation, which allows it to be placed on the anterior face of the shoulder, from the upper surface of the clavicle to the lower part of the coracoid. We leave the guide supported on the base of the coracoid and proceed with the open surgery phase. We make a longitudinal incision of about 6 to 7 cm in the upper part of the clavicle. We open the fascia and deltoid longitudinally and dissect the clavicle from approximately 3 cm medial to the acromioclavicular joint and about 2 cm from the acromion. We resected with an oscillating saw about 4 mm of the distal clavicle and remnants of the meniscus if they still exist. We return to arthroscopic surgery. With the tip of the guide at the base of the coracoid, centered on the medial lateral plane, we place the other end of the guide on the upper part of the clavicle, about 3 cm medial to the acromial edge and centered on the anteroposterior plane. We make a hole with the guidewire and after checking the correct position in the clavicle and coracoid, we drill a tunnel through the 2 cortices of the clavicle and the 2 of the coracoid with a 4.5-cm cannulated drill. Through the cannulated drill, we pass a carrier suture that we use to pass the coracoclavicular suspension implant from the top of the clavicle to the bottom of the coracoid base. We check that the metal tablet is well seated at the base of the coracoid. We place the locking button through the loop of the sutures in the upper part of the clavicle, remove the percutaneous axial traction, reduce the clavicle in both planes, with the help of a periostotome, and pull the sutures. The system locks itself, maintaining the reduction achieved. Once we have the acromioclavicular joint reduced and mechanically fixed with the cortical suspension system, we proceed to add the biological part to the reconstruction. Using an eyelet needle and under arthroscopic control with the scope in the lateral portal, we pass a suture from the anterosuperior part of the clavicle to the inferomedial part of the coracoid. We retrieve this suture from the anterior portal. We pass another suture in the same manner from the posterosuperior part of the clavicle to the inferolateral part of the coracoid. We joint both sutures and obtain a suture that goes from the posterosuperior part of the clavicle, embracing the coracoid at its lower part and again to the clavicle, at its anterosuperior surface. We use this suture as a carrier for the semitendinosus graft. We make a blind tunnel at the lateral border of the clavicle and another at the superior border of the clavicle with a 3.5-mm drill bit. We communicate both tunnels with a bone clamp. We introduce the end of the graft that comes from the lateral part of the coracoid toward the back of the clavicle through the lateral clavicle tunnel to the top of the clavicle. We tighten the graft well and suture it together. We now have achieved the reconstruction of the coracoclavicular ligaments (conoid and trapezoid). With the rest of the graft that we have and that is inserted and fixed in the upper part of the clavicle, we reconstruct the acromioclavicular ligament. We made a tunnel in the acromion with the 3.5-mm drill, from the medial acromion and to its upper part, about 2 cm lateral to the clavicular edge of the acromion. We will pass one of the ends of the graft through the acromial tunnel from medial to superior and we will suture it on itself, applying tension and reducing the clavicle in the horizontal plane. Thus, we achieve horizontal stability with the reconstruction of the ligaments and the superior acromioclavicular capsule. The rest of the graft is sutured on itself, increasing the thickness, stability, and resistance of the acromioclavicular joint. Finally, it is important to perform a good closure of the fascia and muscle plane.
